# Lifestyle and Dietary Factors Associated with the Evolution of Cardiometabolic Risk over Four Years in West-African Adults: The Benin Study

**DOI:** 10.1155/2013/298024

**Published:** 2013-03-12

**Authors:** Charles Sossa, Hélène Delisle, Victoire Agueh, Roger Sodjinou, Gervais Ntandou, Michel Makoutodé

**Affiliations:** ^1^TRANSNUT, WHO Collaborating Centre on Nutrition Changes and Development, Department of Nutrition, Faculty of Medicine, University of Montreal, CP 6128, Succursale Centre-Ville, Montréal, QC, Canada H3C 3J7; ^2^Department of Health Promotion, Regional Institute for Public Health, University of Abomey-Calavi, 01 BP 918 Cotonou, Benin; ^3^United Nations International Children's Emergency Fund, BP 1146 N'Djamena, Chad; ^4^Bioversity International, West and Central Africa, c/o IITA, 08 BP 0932 Cotonou, Benin; ^5^Department of Health and Environment, Regional Institute for Public Health, University of Abomey-Calavi, 01 BP 918 Cotonou, Benin

## Abstract

*Aim*. To assess in adults from Benin changes in cardiometabolic risk (CMR) using both the Framingham risk score (FRS) and metabolic syndrome (MetS) and to examine the effects of diet, and lifestyles, controlling for location and socioeconomic status. *Methods*. Apparently healthy subjects (*n* = 541) aged 25–60 years and randomly selected in the largest city, a small town, and rural areas were included in the four-year longitudinal study. Along with CMR factors, socioeconomic, diet and lifestyle data were collected in individual interviews. A food score based on consumption frequency of four “sentinel” food groups (meat and poultry, dairy, eggs, and vegetables) was developed. Lifestyle included physical activity, alcohol and tobacco use. Education and income (proxy) were the socioeconomic variables. *Results*. Among the subjects with four-year follow-up data (*n* = 416), 13.5% were at risk at baseline, showing MetS or FRS ≥ 10%. The incidence of MetS and FRS ≥ 10% during follow-up was 8.2% and 5%, respectively. CMR deteriorated in 21% of subjects. Diet and lifestyle mediated location and income effects on CMR evolution. Low food scores and inactivity increased the likelihood of CMR deterioration. *Conclusion*. Combining MetS and FRS might be appropriate for surveillance purposes in order to better capture CMR and inform preventive measures.

## 1. Introduction

The burden of noncommunicable diseases such as diabetes and cardiovascular disease (CVD) is rapidly rising in low-income countries [1]. The increasing prevalence of noncommunicable diseases may be partly explained by the on-going nutrition transition process with major changes in diet and lifestyle patterns. These changes are characterized by shifts from traditional diets typically high in fiber and low in fat to westernized diets high in saturated fat, sugar, salt, and processed foods, combined with a more sedentary lifestyle, stress exposure, and less physical activity all of which increase cardiometabolic risk (CMR) factors [2]. Previous studies in sub-Saharan African countries [3] confirmed the relationship between nutrition transition and the increase of CMR factors. In a previous paper, we reported increasing CMR factors over four years in Benin adults who were apparently healthy at onset of study. The four-year incidence rates of abdominal obesity, insulin resistance (based on HOMA), and low HDL cholesterol were, respectively, 10.8%, 30.7%, and 30.2%, and that of the metabolic syndrome (Mets) 9% [4]. However, it was felt that the MetS does not give a proper measure of CMR profile in this population for reasons discussed elsewhere [5, 6]. We therefore examined the Framingham risk score (FRS) along with the MetS. 

On the one hand, the FRS is a recommended tool in clinical practice to estimate a patient's CVD risk [7]. On the other hand, several studies reported that people with MetS, are at increased risk to develop diabetes and CVD [8–10]. Although the FRS was found to be significantly associated with the likelihood of MetS [11], the two risk estimators appear complementary in their ability to assess CMR. For example, due to the cardioprotective blood lipid profile observed in blacks, MetS is underdiagnosed in this population [12]. Furthermore, the diagnosis of MetS provides a dichotomic “yes-or-no answer” and therefore does not properly reflect the continuum of risk associated with MetS [7], whereas FRS considers dyslipidemia, namely, HDL-C and total cholesterol, on a nondichotomous scale. However, the way age is taken into account in the FRS algorithm leads to underestimation of CMR in the youth even those with MetS [13]. Given these, it is possible that a combination of MetS and FRS provides a better estimate of CMR than each tool individually. 

In the present paper, we examine the association of CMR evolution after four years, as measured by combined FRS and MetS, according to diet and lifestyle in Benin adults, controlling for area of residence and socio-economic status. We expected that sedentary lifestyles and less healthy eating patterns would contribute to deterioration of CMR. 

## 2. Methods 

### 2.1. Subjects and Study Design

Details of study design and sampling were published elsewhere [14]. In brief, this longitudinal and observational study included 541 subjects aged 25–60 years (50% women) randomly selected by multistage cluster sampling in the largest city of Cotonou (*n* = 200), the small-size city of Ouidah (*n* = 171), and rural areas surrounding Ouidah (*n* = 170). Subjects enrolled in the study had no prior diagnosis of hypertension, diabetes, or cardiovascular diseases. Following the baseline study on CMR factors, lifestyle, and dietary patterns, subjects were then followed up, with full data collection after two and four years. Subjects diagnosed for high blood pressure or diabetes during the initial study or later remained in the study cohort. Only the 416 subjects (77.0%) who took part in the initial and final assessment are considered in the present paper.

### 2.2. Data Collection Procedure

The study was carried out from 2005 to 2010 in southern parts of Benin. Baseline data on diet and physical activity was collected using 24-hour recalls. Short food frequency and physical activity questionnaires were designed for follow-up. Venous blood samples were drawn after a 12-hour overnight fast. Blood samples were kept on ice and centrifuged within two hours. Plasma or serum was stored at −30°C until analyzed in the biochemistry laboratory of Institute of Applied Biomedical Sciences of Cotonou. 

### 2.3. Study Variables

#### 2.3.1. Anthropometric Parameters

We measured height, weight, and waist circumference (WC) with the subjects in the standing position and light clothing. The average of two separate measures of WC was used in the analyses. BMI status was categorised as follows: underweight <18.5; normal 18.5–24.9; overweight 25–29.9; obese ≥30 [15]. Generic WC cut-off values for abdominal obesity were 80 cm and 94 cm, respectively, for women and men as recommended by the International Diabetes Federation for sub-Saharan Africans in the absence of specific data [16–18]. 

### 2.4. Blood Pressure

Blood pressure was measured using a mercury sphygmomanometer. Systolic and diastolic blood pressure was measured on the right arm of seated subjects after a 10-minute rest. Means of two readings of systolic and diastolic blood pressure were used in the analyses. The interval of time between the first and the second reading was at least 20 minutes. High blood pressure (HBP) was defined as systolic blood pressure (SBP) ≥130 mmHg or diastolic blood pressure (DBP) ≥85 mmHg [16].

#### 2.4.1. Biochemical Parameters

Using appropriate kits from Elitechgroup (Sées, France) and standard colorimetric enzymatic laboratory methods, fasting plasma glucose, and serum concentrations of total cholesterol (TC), high density lipoprotein–cholesterol (HDL-C), and triglycerides were determined. The ratio of TC/HDL-C was computed. Abnormal values were as follows: high fasting glycemia (HFG) (≥5.6 mmol/L); elevated triglycerides (>1.70 mmol/L); and low HDL-cholesterol (≤1.29 mmol/L in women and ≤1.03 mmol/L in men) [16]. The selected cut-offs for high TC/HDL-C were 4.0 for men and 5.0 for women [19].

#### 2.4.2. Metabolic Syndrome

MetS was defined according to the harmonized definition [16]. Any three of the following five CMR risk factors had to be present: abdominal obesity, elevated serum triglycerides, low serum HDL-C, HBP or treatment for hypertension and HFG or diabetes.

#### 2.4.3. Framingham Risk Score

The FRS includes traditional risk factors such as age, sex, smoking status, blood pressure (whether high blood pressure is treated or not), total cholesterol, and HDL-C concentrations levels, and the presence of diabetes. It assesses the risk of a cardiovascular event in the next 10 years [20] and was validated in Africans Americans [21]. The FRS of study participants was computed at baseline and four years later. After computing the points for each risk factor, the absolute risk percentage was calculated and was stratified into two groups: <10% (low risk) and ≥10% (intermediate and high risk). 

#### 2.4.4. Changes in Cardiometabolic Risk

Changes in CMR were assessed at the light of the evolution of both MetS and FRS. Low CMR meant that Mets was absent and that FRS was <10%. CMR was intermediate or high if Mets was present and/or if FRS ≥10%. The evolution of CMR was defined as follows: *favourable:* low risk at both baseline and follow-up, or high or intermediate risk at baseline and low risk at follow-up; *unfavourable or deteriorating:* low risk at baseline and high or intermediate risk at follow-up or high or intermediate risk at both baseline and follow-up. 

#### 2.4.5. Diet

A “sentinel” food consumption score was calculated from a short food frequency questionnaire (SFFQ) developed from repeated 24-hour food recalls conducted at baseline. The SFFQ referred to usual consumption frequency of 10 food groups in the last three months (legumes and nuts; meat; fish; milk and milk products; eggs; vegetables; fruit and fruit juice; sweets; soft drinks; fast food). The selected food groups were those found to be correlated with intake adequacy of one or several micronutrients according to baseline 24-hour food recalls [22]. Consumption frequency went from 0 (never) to 6 (everyday). Details on micronutrient adequacy score were published elsewhere [23]. Based on the SFFQ results, the combination of only four food groups (meat and poultry, dairy products, eggs, and vegetables) had the highest Cronbach's alpha (0.64) and was retained as “sentinel” food consumption score. The correlation between the sentinel food scores measured at T1 and T2 was 0.57 (*P* < 0.001). The sentinel food scores at T2 were grouped in tertiles for the analyses. 

#### 2.4.6. Lifestyle Variables

Lifestyle data used in the analyses in the present report were those collected at follow-up (T2).

#### 2.4.7. Physical Activity

A short questionnaire adapted from WHO STEPwise [24] was used for follow-up. Participants were asked about their usual physical activity over the past three months for transportation, leisure, main occupation, and housework. Participants described their leisure activities and duration (e.g., football, walking, dancing, jogging...) and house chores (cleaning, washing dishes, hand-washing of clothes, cooking, etc.). Active transportation referred to walking or bicycling. The main occupation of participants was also described in order to define the corresponding level of physical energy expenditure. Average duration and frequency (from zero to more than seven times per week) were reported for each activity. Intensity level of activities was expressed in metabolic equivalents (METs). Physical activity was computed as the time spent in vigorous and moderate activities (≥3 METs). According to WHO guidelines for the prevention of chronic diseases [25], we classified subjects as active (≥3 MET, ≥30 min/day) and inactive (≥3 MET <30 min, or <3 MET, any duration). 

Participants were also asked about weekly hours of sedentary behaviour (excluding main occupation and sleep) defined by very low level of energy expenditure (1.0 to 1.5 METs). Typical sedentary activities include sitting quietly, playing on the computer or any board game, driving a car, and watching TV/video [26]. The frequency ranged from none to more than seven times per week. Average duration was self-reported. Total daily sedentary time (in minutes/day) was determined and divided in tertiles for analysis.

#### 2.4.8. Alcohol Intake

Information pertaining to the last three months was collected in personal interviews. Subjects were asked about their habitual drinking patterns based on the STEPwise questionnaire developed by WHO for chronic disease risk surveillance [24]. A standard unit of one drink was used to assist respondents: 1 bottle of beer (33 cl for small bottle or 60 cl for great bottle), 1 glass of wine (10 cl), or 1 shot of distilled spirit (4 cl). The questionnaire items identify alcohol consumption patterns (frequency, quantity) and the type of beverage (local alcohol, wine, beer, etc.) Mean quantity of pure alcohol (in grams per day) was computed based on the amounts, frequency, and alcohol content of the beverages (4.4% for beer, 11,5% for wine and 40% for local distilled alcohol). Results were also grouped in three categories: none, 0 g/day of alcohol per day; Moderate intake, ≤15 g/day for women and ≤20 g/day for men; heavy drinking, >15 g/day for women or >20 g/day for men [27]. 

#### 2.4.9. Smoking

Data was collected based on both current and past smoking habits using the STEPwise questionnaire [24]. Smoking patterns were classified as current, former, or non smokers. Former smokers are those who had stopped smoking since at least six months.

#### 2.4.10. Socioeconomic Variables

Socioeconomic data pertained to education, place of residence, and socio-economic status (SES), which was assessed using a household amenity score as proxy of household income. The items for the household amenity score included type of latrine; paid domestic help; ownership of land, motorcycle, car, television, mobile phone, land line phone, and refrigerator; electricity, water in the house; type of fuel used for cooking; and wall and floor materials. The SES score was computed separately in each location and tertiles were used in analyses. Details of items and coding are available elsewhere [14, 28].

### 2.5. Statistical Analyses

Data was analyzed using SPSS, version 16.0 (SPSS Inc., Chicago, IL, USA). Descriptive statistics are means and standard deviations for continuous variables and proportions for categorical variables. Differences in diet and lifestyle features at follow-up (T2) according to socioeconomic conditions were assessed using an appropriate chi^2^ test or one-way ANOVA with Tukey post hoc test. Relative risk (RR) of deterioration or CMR during the follow-up period was assessed using multiple logistic regression models controlling for baseline age, and sex. All *P* values were two sided, and the significance level was set at *P* < 0.05. 

### 2.6. Ethical Considerations

The study was approved by the Ethics Committee of the Faculty of Medicine, University of Montreal and by the Ministry of Health in Benin. Written informed consent was obtained from each participant before enrolment. Participants with abnormal values were referred to a physician for diagnosis and treatment. The first medical consultation and prescription were paid by the research project.

## 3. Results

### 3.1. Deterioration of Cardiometabolic Risk

CMR factors, MetS, and FRS ≥10% at baseline and at four-year follow-up are given in Table 1. Final prevalence and means of CMR factors were generally higher compared to initial values except for blood pressure which was significantly lower at follow-up. For WC, we observed a downward trend among women and an upward trend in men. MetS was more prevalent in women while Framingham risk score ≥10% was more prevalent in men. Prevalence of MetS and FRS ≥10% increased significantly over the four years in women only. When combining MetS and FRS ≥10%, high CMR was equally prevalent in men as in women (20% at four-year follow-up). 

Incidence rates of MetS and FRS ≥10% over four years were, respectively, 8.2% (67.8% women) and 5.0% (50% women). CMR deteriorated in 21% of subjects, among whom 50.6% were women and 51.7% were from the largest city. Overall, CMR deterioration was more marked with increasing age, going from 10.3% in younger group to 60.9% in subjects aged 45 years and more (data not shown).

### 3.2. Diet, Lifestyle and Socioeconomic Factors

Lifestyle factors and sentinel food scores according to socioeconomic conditions are described in Table 2. Women were as active as men while younger subjects were more active than older ones. Participants in higher income tertile or with higher education and those who lived in the large city were significantly less active than their counterparts. Men drank significantly more alcohol than women and so did participants with higher education. No difference was observed in alcohol intake across income levels and locations, but better educated subjects had significantly higher alcohol consumption than less educated ones. The proportion of smokers (current and former combined) was higher in men, in low income subjects and in subjects with primary or high school education (compared with no schooling). Sentinel food scores were significantly higher in men than in women and in subjects aged 25–35 years than in those aged 45 years and over. Large city subjects and those with higher income level or higher schooling had a significantly higher sentinel food score.

### 3.3. Association of Diet, Lifestyle, and Socioeconomic Factors with Evolution of Cardiometabolic Risk

Relative risks for deterioration of overall CMR over four years according to socioeconomic, lifestyle, and dietary variables are given in Table 3. Two logistic regression models are shown, Model 1 which includes only socioeconomic factors and Model 2 which also includes behavioural parameters. Men were less likely than women to experience a deterioration of CMR, and so did younger subjects compared to older ones. In Model 1, the RR of deterioration of CMR was significantly lower in younger subjects, in those living in the small city compared to the large city, and in the lower income tertile (compared to the upper tertile). Education had no effect. In Model 2, behavioural variables exhibiting a significant association with deterioration of overall CMR were sedentary time, physical activity status, sentinel food consumption, and smoking status. For inactive subjects, the RR of deterioration of CMR was sixfold compared to active subjects. Accordingly, the upper tertile of sedentary time showed a significantly higher RR of deterioration of CMR. The lower sentinel food score tertile was associated with a significantly increased risk of deterioration of CMR compared with higher food scores. We did not find any significant association of CMR change with alcohol consumption, except that in men only, no or moderate alcohol drinking was significantly protective against CMR deterioration compared with heavy drinking (data not shown). In contrast with Model 1, location and income were no longer significant, while male sex became significantly associated with a lower risk of CMR deterioration.

## 4. Discussion

This first longitudinal study in West Africa showed that the four-year incidence of MetS and FRS ≥10% was 8.2% and 5%, respectively, while at baseline, the former affected 8.7% and the latter 7.2% of the subjects. Combining MetS and FRS ≥10%, CMR deteriorated in 21% of subjects. The effects of residence area and income on CMR evolution appeared mediated by diet and physical activity. Being physically inactive exacerbated CMR, while frequent consumption of four “sentinel” foods (meat, dairy, eggs, and vegetables) appeared protective against CMR deterioration. 

### 4.1. Using Both Framingham Risk Score and MetS to Assess Evolution of Cardiometabolic Risk

We used both MetS and FRS to assess CMR and its evolution for two main reasons. First, some CMR factors are not shared, with gender, age, and smoking only in FRS and abdominal obesity and triglycerides in MetS. Secondly, some studies support that MetS and FRS complement each other, for instance in detecting subjects with low grade inflammation [29] and subclinical atherosclerosis [30]. MetS is a stronger predictor of diabetes while FRS appears better at predicting coronary heart disease [10]. Furthermore, MetS failed to identify subgroups at high cardiovascular risk in the short term (8.5 years), unlike the FRS, in Caribbean Indians with blood glucose abnormalities [31]. 

In our study, 21% of subjects displayed a deterioration of CMR over four years of follow-up, while 2.4% apparently improved. Our results showed, and this is an interesting observation, that more women than men had the MetS while the reverse was true for FRS ≥10%. This can be partly ascribed to the inclusion of smoking in the FRS. Assessing CMR using only the MetS, while common, may not reflect the true risk in men as reported by Ford et al. in African Americans [32]. This gender difference is an additional justification for combining the CMR assessment tools. Furthermore, it was reported that African Americans have a higher prevalence of diabetes and CVD than American whites but a lower prevalence of MetS [33]. This “Metabolic Syndrome Paradox” suggests that the MetS is less effective in Black than in white Americans in identifying the risk for diabetes and CVD [34]. Motala et al. [35] suggested that waist circumference cut-offs need to be improved at least in men to allow MetS to capture appropriately CMR in sub-Saharan Africans. 

In our study, CMR deteriorated equally in men and women when MetS and FRS were used in combination. Moreover, the FRS algorithm attempts to weigh the severity of a risk factor (treated versus untreated hypertension, age, different levels of HDL-C, and total cholesterol) to calculate an absolute risk unlike MetS. We need to develop new models where potentially important CMR factors of MetS and FRS will be critically analyzed as continuous variables, without being redundant, allowing to assess the CMR and its severity. For example, in the new model desired, BP, triglycerides, total cholesterol, HDL-C, fasting glucose, and age, will be assessed on a continuous scale while sex, smoking and abdominal obesity will be included in a dichotomous scale. 

### 4.2. Lifestyle, Dietary Factors, and Cardiometabolic Risk Deterioration

Income status and residence area impacted significantly on CMR. However, these variables were no longer significant when lifestyle factors and sentinel food scores were introduced in the logistic regression models. This suggests that income and residence area effects are mediated or explained by behavioural factors. Income is undoubtedly a determinant of consumption of sentinel food groups. Indeed, “sentinel foods” included in the analyses are animal products (and vegetables) [36], known to be less accessible to lower income groups. This may explain the positive association of the food score with socioeconomic status, thereby providing the link between income and CMR [23].Such foods are also more available in the large city than elsewhere, as shown by the significantly higher sentinel food score in the large city. The four food groups included in the SFFQ are similar to those reported by Kennedy et al. [37] in Mali to be key nutrient-dense food groups (dairy, eggs, fruits and dark green leafy vegetables, fish, red meat, legumes and nuts, or their subgroups) for developing proxy indicators of diet quality adequacy based on dietary diversity scores. Of note, consumption of sentinel food groups may be a useful indicator of the quality of diet. However, studies in various settings [37, 38] were too inconsistent as regards “sentinel” food groups to advocate this approach to dietary surveillance in others areas of Benin. Education level did not have a significant effect on CMR evolution although it was positively associated with the “sentinel” food score. It may be that the expected benefit from higher intake of “sentinel” foods was partially cancelled by the more sedentary lifestyle observed in the better educated subjects. Subjects in rural area and small-size city were more active than their urban counterparts, as were subjects with no schooling compared to those of high school level. This is in accordance with Assah et al. [39] who reported that urban compared with rural residence was associated in the Cameroon with lower physical activity and higher prevalence of MetS. In the large cities of Benin, for instance, motorbike-taxis are commonly used for transportation instead of walking or bicycling as in rural areas.

Our results confirm the well-established beneficial effect of physical activity in reducing CMR as reported in several studies [39, 40]. We were able to verify the protective value of physical activity, although we used a questionnaire instead of the doubly labelled water method or the accelerometer to assess physical activity. The important role of physical activity in the prevention of CVD was also reported in an urban population of young and middle aged men in Tanzania (32). 

Regarding sedentary time assessment, we used self-reported data. Gardiner et al. [41] reported that the test-retest reliability for TV viewing is excellent among adults and suggested that in the absence of objective sedentary measurement, self-reported assessment measures may be used as an alternative. Our results were in accordance with observational epidemiological studies that reported that the more time spent being sedentary, the greater the risk of developing the Mets [42], type 2 diabetes, and CVD [43]. Other studies using accelerometer for physical activity record but with arbitrary threshold for sedentary behavior found that vigorous/moderate activity but not sedentary time predicts metabolic risk [44] and insulin resistance [45]. We did not find any independent association between time spent in sedentary activity and deterioration of CMR in contrast with previous studies [42, 46]. Indeed there was a negative correlation (*r* = −0.23, *P* < 0.001) between time spent in sedentary activity and physical activity duration. This means that subjects who were active spent less time in sedentary activity. It should not then be pertinent to study separately physical activity and sedentary activity in this population. However, potential behavioral interventions for prevention of CMR may emphasize promotion of physical activity as well as reduction of sedentary time. In practice, as suggested by Bankoski et al. [42], reducing sedentary time may be achieved by taking brief activity breaks to disrupt prolonged periods of sitting or by increasing movements while sitting. 

The present research work has limitations. Subjects who were available for the last follow-up (*n* = 416) were somewhat different from those missing this last follow-up (*n* = 125), with a significantly higher proportion of missing subjects in the large city, and significantly higher BMI and WC in the retained subjects. However, there were no differences in socioeconomic and behavioural parameters. Furthermore, the study was only conducted in southern parts of Benin and this is a caveat to extrapolation of the findings to other population groups. However, the specific objective connected with the combined use of the MetS and FRS is unaffected by the extent of subject retention and the profile of drop-outs compared to retained subjects. Only four food groups were retained in the SFFQ while other nutrient-dense food groups were not. Strengths of the present study include the population-based dataset and the objective measurement of evolution of CMR using MetS and FRS. 

In conclusion, CMR deterioration is rising among the study population in connection with nutrition transition reflected in diet, physical activity, and other lifestyle components. Combining MetS and FRS may help detect more at-risk subjects. Diet and lifestyle explained the impact of socioeconomic conditions on CMR. Our findings support the beneficial effect of healthy diet and active lifestyle on CMR. Further research on “sentinel” food groups is required, but urgent public health measures are also needed to avert this adverse evolution of CMR. 

## Figures and Tables

**Table 1:** Evolution of prevalence and means of cardiometabolic risk factors during the four-year follow-up period (*n* = 416).

Risk factors	All			Women (*n* = 208)			Men (*n* = 208)		
	Baseline	4 years	*P* ^a^	Baseline	4 years	*P* ^a^	Baseline	4 years	*P* ^a^
Systolic BP^b^ (mmHg)	122.7 ± 15.5	114.6 ± 18.5	**<0.001**	121.7 ± 15.5	114.5 ± 19.3	**<0.001**	123.6 ± 15.5	114 ± 17.7	**<0.001**
Diastolic BP^b^ (mmHg)	76.5 ± 10.3	70.8 ± 11.9	**<0.001**	75.9 ± 9.4	70.7 ± 12.2	**<0.001**	77.0 ± 11.0	70.8 ± 11.7	**<0.001**
High BP^c^	35.1	21.6	**<0.001**	19.5	14.9	0.151	18.3	11.1	**0.037**
FG^d^ (mmol/L)	4.8 ± 0.6	5.0 ± 1.0	**<0.001**	4.8 ± 0.7	5.0 ± 1.1	**0.039**	4.8 ± 0.6	5.0 ± 0.8	**0.002**
High FG^e^	9.6	20.0	**<0.001**	10.1	17.3	**0.032**	9.1	22.6	**<0.001**
HDL-C (mmol/L)	1.4 ± 0.4	1.3 ± 0.5	**<0.001**	1.5 ± 0.4	1.3 ± 0.4	**<0.001**	1.4 ± 0.4	1.3 ± 0.5	**0.023**
Low HDL-C	26.2	37.7	**<0.001**	31.2	47.1	**<0.001**	21.2	28.4	0.088
TC/HDL-C	3.2 ± 1.1	3.7 ± 1.5	**<0.001**	3.1 ± 1.0	3.7 ± 1.5	**<0.001**	3.4 ± 1.2	3.7 ± 1.6	**<0.001**
High TC/HDL	13.7	22.6	**<0.001**	18.8	28.8	**0.015**	8.7	16.3	**0.017**
TG (mmol/L)	0.7 ± 0.4	1.0 ± 0.6	**<0.001**	0.7 ± 0.3	0.9 ± 0.5	**<0.001**	0.8 ± 0.5	1.0 ± 0.7	**<0.001**
High TG	2.2	8.7	**<0.001**	1.0	8.7	**<0.001**	3.4	12.0	**<0.001**
WC (cm)	85.4 ± 12.5	84.7 ± 12.0	**0.034**	87.9 ± 13.4	85.6 ± 12.9	**<0.001**	82.9 ± 11.0	83.7 ± 11.2	**0.038**
Abdominal obesity	42.5	42.3	0.944	69.2	65.9	0.463	15.9	18.8	0.436
Body mass index	24.3 ± 6.5	25.1 ± 5.7	**<0.001**	26.0 ± 6.1	27.0 ± 6.3	**<0.001**	22.5 ± 4.1	23.2 ± 4.2	**<0.001**
Overweight (%)	23.8	24.3	0.871	30.8	26.0	0.276	16.8	22.6	0.139
Overall obesity (%)	7.2	11.3	**0.041**	22.1	35.1	**0.003**	5.8	7.2	0.550
MetS (%)	8.7	13.9	**0.015**	11.1	18.8	**0.027**	6.2	9.1	0.269
FRS ≥ 10%	7.2	11.3	**0.041**	2.4	6.7	**0.034**	12.0	15.9	0.257
MetS and FRS ≥ 10%	2.4	4.3	0.124	1.4	4.3	0.078	3.4	4.3	0.610
MetS or FRS ≥ 10%	13.5	20.9	**0.004**	12.0	21.2	**0.012**	14.9	20.7	0.123

Values are expressed as means ± SD or percentage.

BP: blood pressure, FG: fasting glycemia, HDL-C: high density lipoprotein cholesterol, TC: total cholesterol, WC: waist circumference, TG: triglycerides, MetS: metabolic syndrome, FRS: framingham risk score.

^a^ *P*: for *t*-test or *χ* ^2^ test.

^b^33 subjects (23 women and 10 men) under medical treatment for high blood pressure are excluded.

^c^Includes 33 subjects (23 women and 10 men) under medical treatment for high blood pressure.

^d^4 subjects (1 woman and 3 men) under medical treatment for diabetes are excluded.

^e^Includes 4 subjects under treatment for diabetes.

**Table 2:** Lifestyle features and dietary indicator at last follow-up according to socioeconomic conditions (*n* = 416).

	Physical activity					
	Vigorous or moderate activity (mn/day)	Active %	Sedentary time (mn/day)	Alcohol intake (g/day)	Current or former smokers %	Food score
Sex						
Women	166.0 ± 141.2	74.0	88.3 ± 83.7	2.3 ± 5.3	1.0	3.9 ± 4.6
Men	176.2 ± 168.5	75.5	115.2 ± 97.2	9.4 ± 13.6	26.0	5.2 ± 4.9
*P*	0.503	0.735	**0.003**	**<0.001**	**<0.001**	**0.010**
Age						
25–34	198.8 ± 160.1^a^	83.9^a^	106.0 ± 96.5^a^	6.9 ± 13.4	10.9	5.3 ± 4.9^a^
35–44	156.2 ± 154.2^b^	68.9^b^	94.3 ± 85.7^b^	5.6 ± 9.7	15.6	4.3 ± 4.6^ab^
≥45	146.1 ± 144.0^b^	67.5^b^	103.2 ± 90.4^b^	4.5 ± 7.5	15.0	3.7 ± 4.7^b^
*P*	**0.007**	**<0.001**	0.549	0.160	0.433	**0.023**
Study sites						
Rural area	194.6 ± 148.5^a^	85.3^a^	30.8 ± 51.5^a^	5.8 ± 10.6	10.9	3.0 ± 4.2^a^
Small city	198.1 ± 158.1^a^	80.0^ a^	48.5 ± 65.7^a^	6.2 ± 12.1	14.2	3.4 ± 3.9^a^
Large city	127 ± 150.5^b^	58.3^b^	73.2 ± 78.05^b^	5.6 ± 9.6	15.2	7.4 ± 5.0^b^
*P*	**<0.001**	**<0.001**	**<0.001**	0.886	0.56	**<0.001**
Income score						
Low	216.9 ± 160.7^a^	87.2^a^	71.8 ± 67.3^a^	5.6 ± 10.3	19.1^a^	3.1 ± 4.0^a^
Medium	180.8 ± 160.4^a^	73.8^b^	107.9 ± 93.9^b^	5.4 ± 8.8	9.7^b^	5.6 ± 5.3^b^
High	110.7 ± 121.6^b^	62.3^c^	127.4 ± 102.8^b^	6.8 ± 13.4	11.5^b^	5.0 ± 4.7^b^
*P*	**<0.001**	**<0.001**	**<0.001**	0.590	**0.047**	**<0.001**
Education						
No schooling	199.4 ± 166.7^a^	76.2^ab^	68.7 ± 68.36^a^	3.2 ± 7.1^a^	6.7^a^	3.1 ± 4.1^a^
Primary school	185.5 ± 151.4^a^	81.4^b^	97.2 ± 84.4^b^	5.1 ± 8.6^a^	17.2^b^	4.0 ± 4.5^a^
High school	140.7 ± 146.8^b^	68.2^a^	126.6 ± 103.1^c^	8.2 ± 13.9^b^	14.5^b^	5.9 ± 5.1^b^
*P*	**0.004**	**0.045**	**<0.001**	**0.001**	**0.048**	**<0.001**

Values are expressed as mean ± SD or percentage.

*P* for *t*-test or *χ* ^2^ test one way or ANOVA.

^a,b,c^Mean values with unlike superscript letters were significantly different (*P* < 0.05; oneway ANOVA test with Tukey post hoc).

**Table 3:** Relative risk for deterioration of cardiometabolic risk factors over the follow-up period after adjusting for age and sex (*n* = 416).

Variables	Deterioration of cardiometabolic risk	
	Model 1 RR (CI 95%)	Model 2 RR (CI 95%)
Sex		
Women	1	1
Men	0.93 (0.51–1.67)	**0.41 (0.18-0.95**)
Age (years)		
≥45	1	1
25–34	**0.06 (0.03–0.14)**	**0.40 (0.01–0.11)**
35–44	**0.29 (0.16–0.53)**	**0.14 (0.06–0.33)**
Location		
Large city	1	1
Rural area	0.56 (0.27–1.18)	0.54 (0.19–1.53)
Small city	**0.39 (0.21–0.74)**	0.40 (0.16–1.02)
Income score		
High income	1	1
Low income	**0.42 (0.19–0.92)**	0.57 (0.2–1.63)
Medium income	0.75 (0.39–1.44)	1.18 (0.51–2.7)
Education		
High school	1	1
No schooling	0.63 (0.27–1.47)	0.53 (0.17–1.66)
Primary school	1.05 (0.53–2.11)	1.21 (0.48–3.05)
Sedentary time		
High tertile		1
Low tertile		**0.04 (0.01–0.13)**
Medium		**0.24 (0.11–0.55)**
Physical activity		
Active^a^		1
Inactive		**6.29 (2.97–13.35)**
Food score		
High tertile		1
Low tertile		**5.61 (1.91–16.41)**
Medium tertile		2.02 (0.80–5.07)
Alcohol intake		
Heavy drinking		1
None		0.51 (0.16–1.64)
Moderate drinking		0.72 (0.25–2.06)
Smoking		
Current smoking		1
Non smoker		**0.14 (0.02–0.85)**
Former smokers		0.34 (0.05–2.29)

Model 1: Income score + location + education level.

Model 2: Income score + location + education level + healthy food score + lifestyle components.

^a^Inactive: <30 min/d moderate/vigorous activity, active: ≥30 min/d moderate/vigorous activity.
